# New Rootsnap Sensor Reveals the Ameliorating Effect of Biochar on *In Situ* Root Growth Dynamics of Maize in Sandy Soil

**DOI:** 10.3389/fpls.2020.00949

**Published:** 2020-06-25

**Authors:** Fauziatu Ahmed, Emmanuel Arthur, Hui Liu, Mathias Neumann Andersen

**Affiliations:** ^1^ Regional Office for Africa, Food and Agriculture Organization of the United Nations, Accra, Ghana; ^2^ Department of Agroecology, Faculty of Technical Sciences, Aarhus University, Tjele, Denmark; ^3^ Department of Crop Production Ecology, Swedish University of Agricultural Sciences, Uppsala, Sweden

**Keywords:** maize root, straw biochar, subsoil, *in situ* root method, drought stress

## Abstract

We investigated if subsoil constraints to root development imposed by coarse sand were affected by drought and biochar application over two seasons. Biochar was applied to the subsoil of pots at 20–50 cm depth in concentrations of 0%, 1%, 2%, and 3% (B0, B1, B2, and B3). Maize was grown in the same pots 1 week and 12 months after biochar application. The maize plants were fully irrigated until flowering; thereafter, half of them were subjected to drought. A new method for observing root growth dynamics and root length density *in situ*, the Rootsnap sensor system, was developed. The sensors were installed at 50 cm depth just below the layer of biochar-amended subsoil. Using data from a smaller experiment with grass, the calculated root length densities from the sensors were compared with data from scanning of manually washed roots. In year 2, we investigated the effect of aged biochar on root growth using only the root wash and scanning method. The Rootsnap sensor revealed that the arrival time of the first root in B3 at the 50 cm depth averaged 47 days after planting, which was significantly earlier than in B0, by 9 days. The tendency for faster root proliferation in biochar-amended subsoil indicates that biochar reduced subsoil mechanical impedance and allowed roots to gain faster access to deep soil layers. A linear regression comparing root length density obtained from the Rootsnap sensor with the scanning method yielded an *r* ^2^ of 0.50. Our analysis using the scanning method further showed that under drought stress, maize roots responded with reduced root diameter and increased root length density at 50–70 cm depth in the first and second year, respectively. The trend under full irrigation was less clear, with significant decrease in root length density for B1 and B2 in year 2. Overall, reduction in subsoil mechanical impedance observed as early arrival of roots to the subsoil may prevent or delay the onset of drought and reduce leaching of nutrients in biochar-amended soil with positive implications for agricultural productivity.

## Introduction

The plant root is a principal water absorbing organ, that plays a crucial role in the development of plants, especially when water is limited (Upchurch and Ritchie, 1983). Under drought conditions, dehydrating roots synthesize abscisic acid (ABA), which is transported to the shoot to signal the level of drying in the soil (Zhang and Davies, 1989). This results in the partial closure of stomata to prevent water loss (Zhang and Outlaw, 2001) and consequently decrease photosynthesis, often to a smaller degree, thus enhancing water use efficiency (Liu et al., 2005). In maize, the accumulation of ABA results in the maintenance of root elongation and inhibition of shoot elongation at low water potentials (Saab et al., 1990). This is an example—out of many—of how abiotic stress influences root system architecture (Koevoets et al., 2016) as well as shoot growth.

Quantification of plant root geometry, in particular parameters such as root length, root length density or root diameter, is pivotal to understand many plant physiological functions (Pierret et al., 2013) and responses under stress conditions. Most plant studies have, however, focussed on aboveground parameters because root studies are quite cumbersome and labour intensive. Over the years, several methods have been developed and used for root assessment such as soil coring, trench wall and root mapping techniques, core break, minirhizotron, pinboard, excavation, and washing methods (Smit et al., 2000). In samples obtained from soil coring, roots can be washed free of soil and analyzed with software such as the WinRHIZO (Regent Instruments Inc., Canada) after scanning or the length can be estimated with the Newman (1966) counting method. Alternatively, the core-break method may be employed by breaking the retrieved soil core at the depth of interest (Bohm, 1979). Roots visible from the broken cross-section are counted with the naked eye and the root intensity calculated using the cross-sectional area of the soil core. If perpendicular sides are counted, it is possible to calculate root length densities directly (Chopart and Siband, 1999). The trench wall and pinboard techniques, utilize exposed roots from an evacuated plane, the former involves plotting root ends to a transparent sheets and the latter, a board containing nails arranged in a grid that is washed to expose roots (Smit et al., 2000). All the methods mentioned above with the exception of the minirhizotrons are destructive, which poses a challenge or makes it impossible to observe root development over time.

Sandy soils are characterised by low water holding capacity and excessive drainage below the root zone (Andry et al., 2009) and therefore considered marginal for agricultural production especially in dry areas. Nevertheless, many hundred thousands of hectares of this soil type formed by glacial river deposits is under cultivation in north-western Europe (Andersen and Aremu, 1991; Andersen et al., 1992; Ahmed et al., 2018). In coarse sand, lack of soil structure, low moisture content, greater bulk density and low organic content tend to increase the mechanical impedance to root growth (Bruun et al., 2012; Wernerehl and Givnish, 2015). Over the past few years, there have been efforts to enhance productivity of sandy soils by applying biochar, a carbon rich product which is produced by heating biomass to above 250°C in the absence of or with limited oxygen (Lehmann and Joseph, 2015). Several studies have reported improvement in soil water content and root development, decrease in bulk density, pore volume, and nitrate leaching (Abel et al., 2013; Bruun et al., 2014; Abiven et al., 2015; Haider et al., 2017) of sandy soils amended with biochar. Literature describing biochar’s influence on maize growth have mostly reported on aboveground biomass (Major et al., 2010; Uzoma et al., 2011; Zhang et al., 2012; Sänger et al., 2017) with only few studies on roots. In tropical sandy soil, Abiven et al. (2015) reported an enhancement of maize root traits such as biomass and surface area with corncob biochar application. In temperate soil, some studies (Prendergast-Miller et al., 2011; Bruun et al., 2014; Ventura et al., 2014; Hansen et al., 2016) have reported effects of biochar on root development; however, studies on maize roots in temperate soils are limited. Although use of fresh biochar dominates research, biochar’s effects may vary over time due the oxidation of carboxylic groups on the edges of the aromatic backbone (Glaser et al., 2000) and gradually change soil properties. Hence, its effect on roots may vary as biochar ages in the soil.

In this paper, we investigated the effect of wheat straw biochar on maize subsoil root development and response under drought conditions using a new method for observing roots *in situ* and dynamic changes in root density distribution. The details on how to assemble the Rootsnap sensor, aimed at providing an accurate and cost-effective way of analysing roots growth dynamics are discussed. We assessed whether root length density estimates from the Rootsnap sensor are comparable with conventional methods of soil sampling, separation of roots from soil by washing and measuring root length. Lastly, we investigated the effect of biochar on root development under full irrigation and drought with fresh and aged biochar amendment of the subsoil.

## Materials and Method

### Rootsnap Sensor

**Figure 1:**
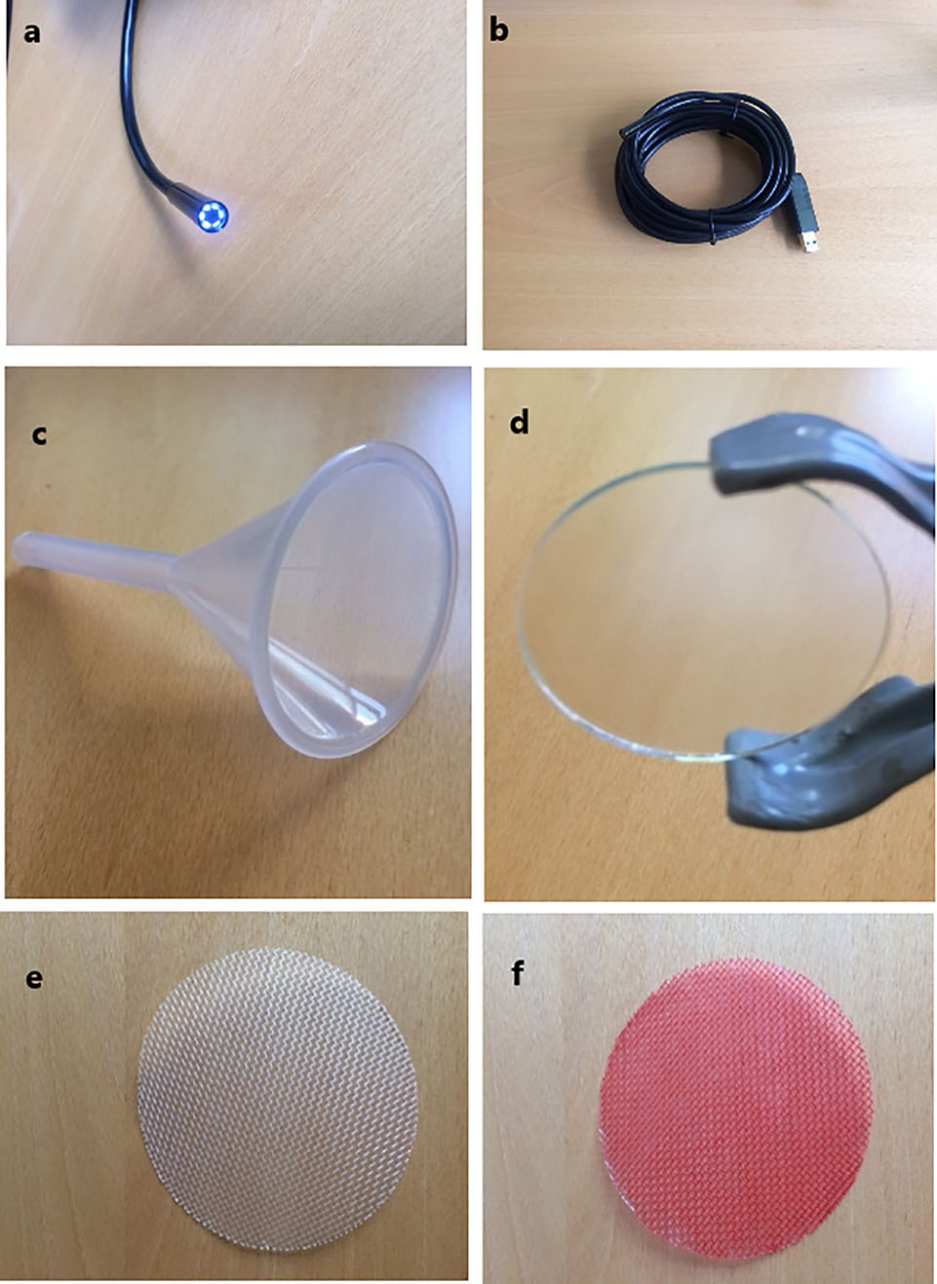
Rootsnap sensor components. **(A)** Imaging device showing the lens with diameter of 7 mm; **(B)** entire endoscope with cable and usb connector; **(C)** frame component showing the funnel with diameter of 6.0 cm; **(D)** antiglare glass with diameter of 4.5 cm; **(E)** white nylon mesh with diameter of 6.5 cm; **(F)** red coloured nylon mesh.

The Rootsnap sensor consists of an imaging device (**Figures 1A, B** **)** for taking pictures and videos, a frame component (**Figures 1C, D** **)** and the root-counting component (**Figures 1E, F** **)**. The imaging device is a waterproof standard endoscope (Shenzhen FDL Technology co, PRC) with an image capture resolution of 640 × 480. The lens (**Figure 1A**) is surrounded by six white LED lights, which provide light controlled by an adjustable light switch. The device is powered through the USB of a computer or external USB by a cable, which can be either 2, 5, or 10 m (**Figure 1B**). The view angle of the camera is 66°. The camera and its housing case have a diameter of approximately 7 mm. The frame comprises of a plastic funnel and anti-glare glass (Hengshi Aohong International, PRC). The funnel’s conical mouth is 6 cm in diameter at its widest point; the stem is 5.5 cm long and 0.8 cm in diameter. The funnel holds the endoscope and the other components together. It also holds silica gel, which protect the lens from condensation. The anti-glare glass is 4.5 cm in diameter and it functions to prevent roots from growing directly on the lens. The root-counting component is a nylon wire mesh (Shanghai bolting cloth manufacturing, PRC) of diameter 9 cm with openings of 743 micron. Roots intersecting with the mesh in the plane of observation were counted for determining the root length density. The nylon mesh, which comes originally in white, **Figure 1E** was coloured red **Figure 1F** to enhance contrast.

#### Assembling the Rootsnap Sensor

**Figure 2:**
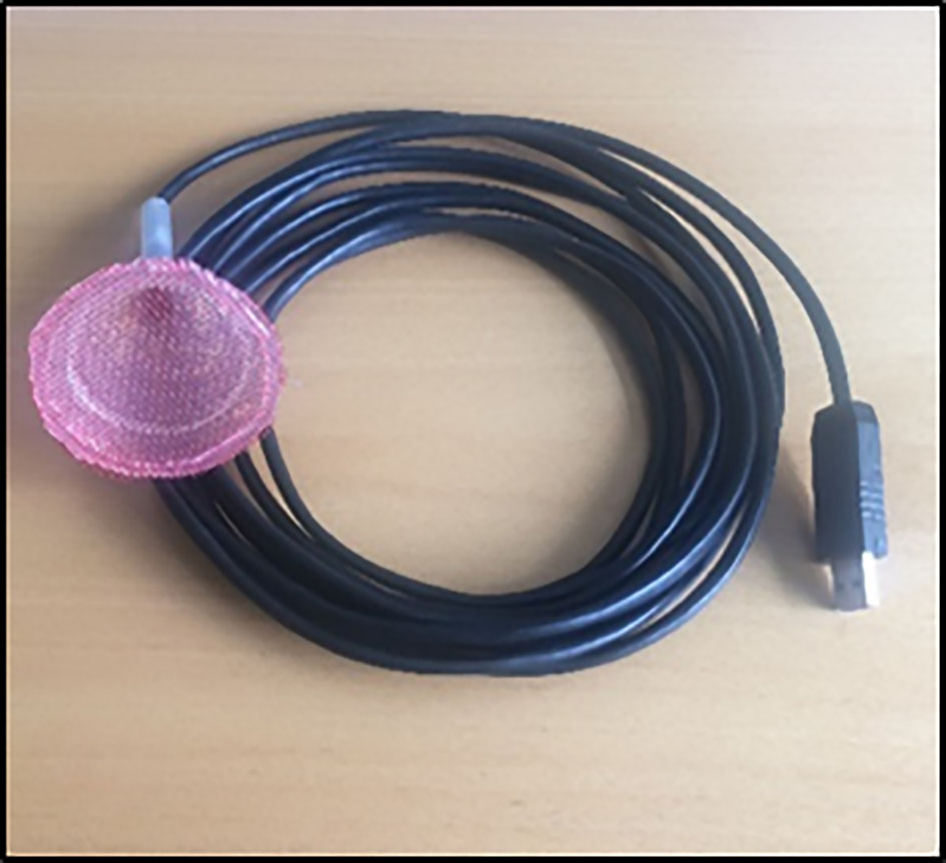
Assembled Rootsnap sensor.

The tip of the endoscope camera was coated carefully with epoxy rapid glue to avoid glue sticking on the lens. It was then placed in the funnel through the stem and allowed to set for an hour. The stem of the funnel fitted with the endoscope was filled with a neutral silicon sealant and allowed to set for 3 days. A clamp was used to hold the set up together and glue was applied to the sides of the funnel about 1 cm from the rim. The anti-glare glass was placed in the funnel and allowed to set for a day. The wire mesh was placed on the rim of the funnel and melted silicon was applied to attach the mesh to plastic funnel. The excess mesh was trimmed after the silicon had solidified. The assembled root sensor is presented in **Figure 2**.

### Experimental Set Up

#### Maize Experiment

**Figure 3:**
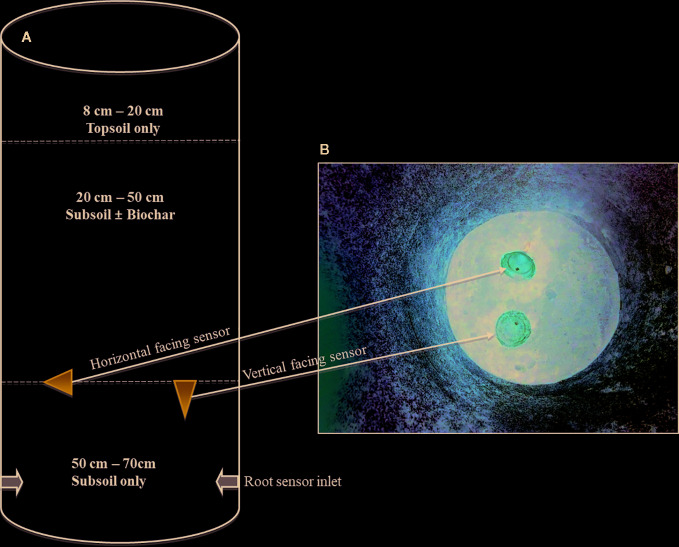
Diagram of the pot set-up **(A)** and illustration of the sensors positions at 50 cm **(B)**.

The study was conducted at Research Centre Foulum of Aarhus University (AU-Foulum), Denmark. The research was part of an experiment reported by Ahmed et al. (2018), who described the setup and results on aboveground growth and physiology of maize. Sand-textured soil was sampled from the Jyndevad research station at depths of 0–25 cm and 25–100 cm to represent topsoil and subsoil, respectively. Further information on the soil textural composition can be found in Ahmadi et al. (2010). The pots that were used had diameter of 36 cm and height of 70 cm. Prior to filling the pots, the inside was coated with subsoil mixed with water insoluble wallpaper glue (Bostik Hernia Vaadrumslim) as done by Bruun et al. (2014) to prevent preferential root growth along the sides of the pot. The preparation of the pots started with placing two Rootsnap sensors in the pots with their USB-cable passing through an opening in the side of the pot. The cable with the USB-connector part of the sensor was connected to a USB hub. The part with the lens was inside, and initially placed on the top edge (opening) of the pot. Prior to filling the pots, samples of soil and biochar were both oven dried to measure the water content. This was used to determine the desired dry weight based proportions, which were then mixed together for 5 min in a mechanical mixer. The soil was packed to a bulk density of 1.2 g/cm^3^ and 1.3 g/cm^3^ in the topsoil and subsoil sections, respectively. Packing was done by pressing the soil down with fingers to marks placed every 10 cm inside the pots followed by surplus irrigation and allowing the soil to settle. A diagram of the pot setup is presented in **Figure 3A**. After filling the 50–70 cm of the pots with subsoil, the USB-connector end of the cable was then pulled to position the sensors at the 50 cm depth. One was facing vertical and the other facing sideways as shown in **Figure 3B**. To prevent soil from subsequent layers to go through the mesh, a thin layer of water-soluble wallpaper glue was brushed on the surface of the vertical sensor. Thereafter sandy subsoil ( ± biochar) was filled in the 20–50 cm depth by slowly placing soil and then pressing gently with the fingers to avoid soil going through the mesh of the vertical sensor. This procedure may successfully be replaced—where convenient—by placing thin plastic foil over the mesh, and this foil is then later removed by carefully pulling it out. Finally, the topmost layer in all pots was filled with sandy topsoil at the depth of 8–20 cm in the pots.

The treatments thus comprised biochar applied at concentrations of 0, 1, 2, and 3% (B0–B3) and irrigation at two levels. Wheat straw biochar used for the experiment was produced by slow pyrolysis at a temperature of 600°C (Frich A/S, Denmark). Biochar properties are shown in Ahmed et al. (2018). The plants were initially irrigated each time the water deficit exceeded 25% of the available water content. At tasselling, pots of each biochar treatment were assigned to one of two groups. In one of the groups, irrigation was continued as described (FI) while the second group of pots was subjected to two drying cycles (D). This resulted in a total of 64 pots (eight replicates per treatment) of which 32 pots had the Rootsnap sensors (64 in total) installed. The treatment combinations are shown in **Table 1**.

**Table 1:** Treatment combinations.

Biochar concentration (%)	Irrigation Level	Designation
0	Full	B0FI
0	Drought	B0D
1	Full	B1FI
1	Drought	B1D
2	Full	B2FI
2	Drought	B2D
3	Full	B3FI
3	Drought	B3D

Maize was sown on 7^th^ May 2015 and 20^th^ April 2016 in the first and second year, respectively. In both years, three seeds were planted, and then thinned to one plant per pot after emergence. The growing conditions in the greenhouse was a maximum day temperature of 28°C and a night temperature of 10°C with no artificial lightening. The plants were harvested at 104 days after planting (DAP) in 2015 and 111 DAP in 2016. The 32 pots with Rootsnap sensors installed were destructively sampled for bulk density and root length density analysis in 2015. Samples for root determination were taken between 45–50 cm soil layer. The remaining 32 pots without sensors were kept for the second year experiment and destructively sampled at the end of year 2 experiment.

##### Grass Experiment

A supplementary grass experiment was conducted at the same location as the maize experiment from 4^th^ November to 6^th^ December 2015 with 14 pots of dimensions, 21 cm height and 26 cm in diameter. There were no treatments in this experiment. The Rootsnap sensors were installed at 5–10 cm depth in pots filled with sandy loam soil. Grass turf consisting of a mixture of ryegrass (*Lolium perenne* L.) and red fescue (*Festuca rubra* L.) was transplanted to the surface of the soil. The grass was subjected to the same growth conditions as the maize. Irrigation to field capacity was carried out every 3 days.

### Measurements

#### Data From Rootsnap Sensor

The Rootsnap sensors were connected to a computer *via* a USB hub. A timer was used to designate times for root images to be taken automatically with a streamer software on a Linux operating system. Images were taken six times per day. When it was time for image capture, the timer switched on electricity for 15 min. During that period, the cameras took a few rounds of images and then the system shut down—including the camera light—until the next scheduled session.

The arrival time of roots at the 50 cm depth was used to determine the ease of movement of roots through the biochar amended subsoil. This was apparent from the root images taken late in the season by capturing the date and time a root had made its way through the above soil layers and the root tip just penetrated the mesh of the sensors. Images were collected into time-lapse videos to give a general overview of the dynamics of root development for the entire experimental period.

The number of roots intersecting with the mesh (n) was counted and the time of each intersect noted. The number of root per square cm (N) was determined by dividing the root count by the area of the field of view (12 cm^2^). For the vertical facing camera, this was labelled N_V_ and for the horizontal facing camera N_H_.

The average of root intersects per cm^2^ for the two cameras were calculated as:

1 $$N_{A}=\frac{N_{V}+N_{H}}{2}$$

Anisotropy (A) accounts for the non-uniform directional distribution of roots, which specifically is related to root gravitropism (e.g., Xiao and Zhang, 2020) and more generally results from both morphogenetic and environmental factors interacting during the development of the root systems (Smit et al., 2000). The extent of this phenomenon was calculated using Eq. 2 (Van Noordwijk, 1987; Chopart and Siband, 1999):

2 $$A=ABS\frac{\left(1-\frac{N_{V}}{N_{H}}\right)}{\left(2\times \frac{N_{V}}{N_{H}}+1\right)}$$

The root length density (L_V,_ cm cm^-3^) was then calculated according to Chopart and Siband (1999) using Eq. 3.

3 $$L_{V}=2\times N_{A}(0.5\times A^{2}+1)$$

The results of the root length density of maize and grass obtained from the Rootsnap sensor was compared to results obtained from scanning manually washed roots.

#### Soil Samples

In 2015, the 32 pots of maize containing the Rootsnap sensors were excavated after harvest for sampling. Soil samples for determination of bulk density and soil water content were extracted using sampling cores of 100 cm^3^. Thereafter, loose soil-root samples of c. 1 kg were taken with a sharp shovel from depths of 20–50 cm and 50–70 cm. In 2016, the remaining 32 pots were excavated and soil-root samples were extracted only from the 50–70 cm layer. For the grass experiment, samples were taken from the 5–10 cm depth. The samples were weighed and stored in a freezer at -18 °C until roots were extracted. Soil bulk density and the volumetric water content of the soil was obtained by weighing 100 cm^3^ samples obtained by the core method, before and after oven drying the samples at 105°C.

#### Root Extraction and Scanning

The frozen soil samples were allowed to thaw in a 10°C room overnight prior to root washing. Samples were divided with a sample divider and the amount to be washed was weighed. The soil-root sample was mixed with water and stirred in a bucket until the soil was fully dispersed. The supernatant was decanted into a 0.5 mm sieve stacked on top of the 0.25 mm sieve. This was repeated several times until there were no more visible roots floating in the bucket. The content of the 0.5 mm sieve was transferred to a white photo tray leaving the sediment. Debris and biochar particles were removed using tweezers. White live roots were separated from dead ones. Roots that were dark and sank to the bottom were assumed dead and probably derived from previous crops. The same was repeated with the content of 0.25 mm sieve. All live roots were mixed with 3% acetic acid and poured into bags for freezing. Prior to scanning, roots were thawed and stained using a neutral red solution comprising of 0.5 g “neutral red” dissolved in 100 ml 96% ethanol and 900 ml distilled water. The roots were soaked in the staining solution for 24 h while stored in a refrigerator at 5°C. The surplus colour was removed by rinsing with demineralized water before scanning. The resolution of the scanner was set to 600 dpi. After scanning, the analysis of the image was done with the WinRHIZO software (Regent Instruments Inc., Canada). The analysis of the root length was done by excluding the two lowest diameter classes of 0<L ≤ 0.05 mm and 0.05<L ≤ 0.10 mm, which were considered to be mycorrhiza. The root length was divided by the volume of soil to obtain the root length density. The average diameter of roots at the 50–70 cm depth of maize plants was calculated by summing up the product of the root length in each class and the average diameter of that class and dividing by total root length.

#### Newman Counting

To analyse the effect of biochar on RLD in the subsoil biochar layer in 2015, root samples from the 20–50 cm depth were analysed using the Newman (1966) counting method for the control (B0) and the highest biochar concentration (B3) under both FI and D. The reason for using this procedure was that these samples were too cumbersome to clean sufficiently for scanning and subsequent image analysis. Ten maize root samples, which had been washed and analysed using the scanning method were re-analysed using the counting method. The washed roots were poured into a 0.25 mm sieve and placed in a tray with water forming a thin film over the mesh. Then the roots were carefully spread to make an even distribution over the area of the sieve. With a binocular microscope, the number of intersections between roots and a horizontal hairline placed in the microscope’s eyepiece were counted. The root length, R was determined using Eq. 4, Newman (1966).

4 $$R=\frac{\pi NA}{2H}$$

Where N is the number of intersections, A is the area of the sieve, H is the total length of the lines provided by the hairline of the microscope.

### Statistical Analysis

Statistical analysis was conducted with SigmaPlot 11 (Systat Software, San Jose, CA). One and two way Analysis of Variance (ANOVA) was used to analyse the effect of biochar and irrigation. The Holm-Sidak posthoc test was used to compare treatments. P values of less or equal to 0.05 was taken to indicate statistical difference.

## Results

### Images and Videos From the Rootsnap Sensor

**Figure 4:**
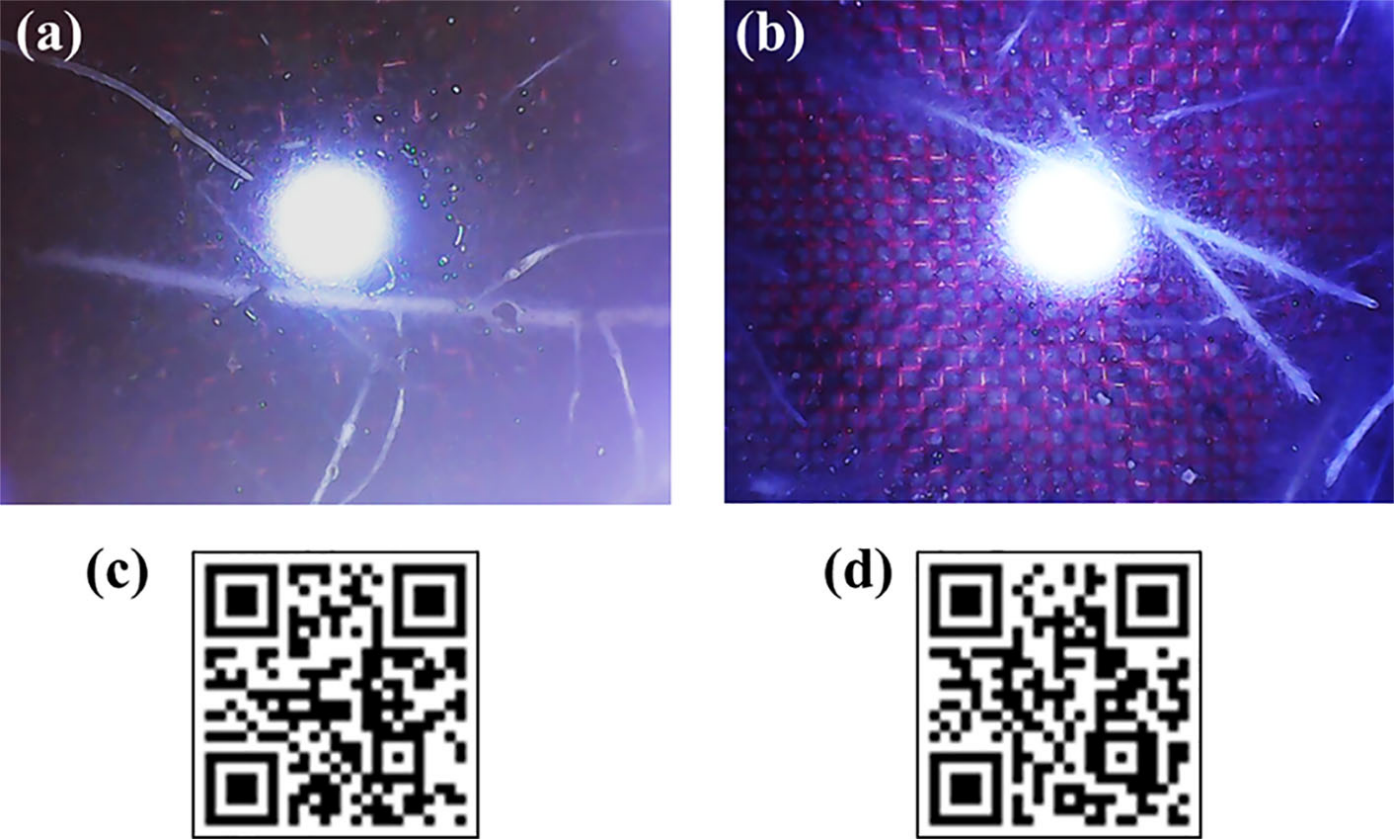
Root images of maize **(A)** and grass **(B)**, and QR codes for root videos of maize **(C)** and grass **(D)**. The openings in red nylon wire mesh shown in a and b are 0.743 mm.

**Figure 5:**
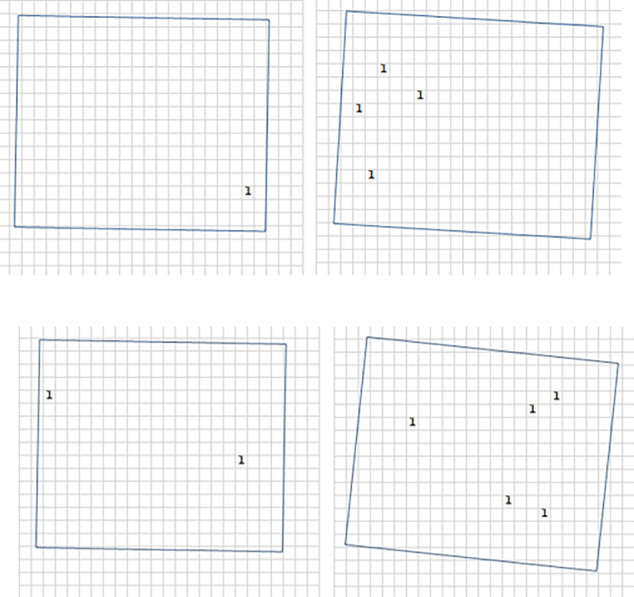
Root distribution in field of view of the Rootsnap sensors for control (B0; top) and biochar 3% (B3; bottom). For counting and registering the position of the roots, an Excel sheet mimicking the nylon mesh (see **Figures 4A, B**) was created. The number and position of roots intersecting with the mesh was counted and the time of each penetration of the mesh by a root tip was noted.

The newly developed Rootsnap sensor was successfully used to automatically capture images of maize and grass roots while they were growing in soil. The images were stored on a computer and were made accessible remotely *via* internet connection. Examples of images obtained from the root sensor are shown in **Figures 4A, B** for maize and grass, respectively. Some of the root videos from the maize and grass experiment can be viewed by scanning the QR codes **Figures 4C, D** (QR codes reader can be downloaded online for both IOS and android mobile and PC devices). The pictures obtained were processed manually by looking through the sequence. Each time a new root tip penetrated the mesh of a sensor, the position and time was noted in an Excel sheet, which mimicked the mesh. An example of this is shown in **Figure 5**.

### Effect of Biochar on Root Penetration to Subsoil Layer

**Figure 6:**
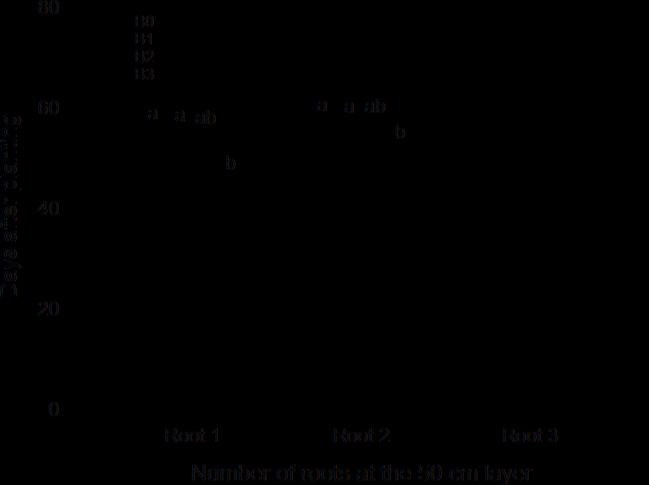
Day of arrival of first, second and third root to the 50 cm depth after passing the subsoil layer with different biochar levels. Values are averaged over irrigation treatments. Error bars indicate standard errors of the mean (n=5(B0), 3(B1), 3(B2), 5(B3)). Different letters indicate significant difference. Letters are not shown when there is no significant difference.

Biochar tended to ease the downward growth of roots in coarse sandy soil as opposed to the control, which restricted root penetration. The arrival time of the first root in B3 was significantly (P ≤ 0.006) earlier than for B0 and B1 treatments (**Figure 6**). A similar observation was made for the mean arrival time of the second root (P ≤ 0.016). The delay of first control (B0) root to arrive at the 50 cm layer by up to 9 days may have implications for crop growth in terms of access to water especially during drought.

### Comparison of Methods With Root Length Density Estimates

**Figure 7:**
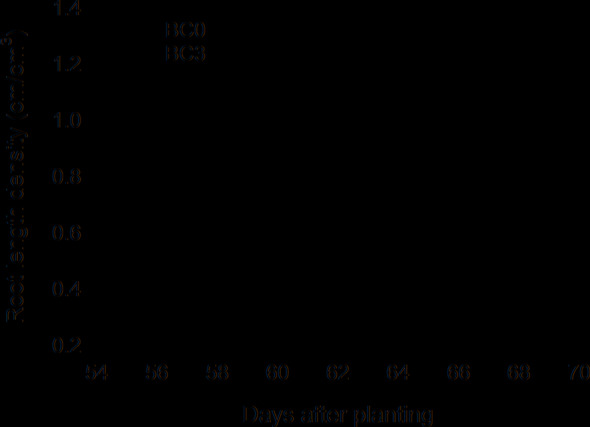
Root length density over time for control (B0) and biochar 3% (B3) averaged over irrigation treatments.

**Figure 8:**
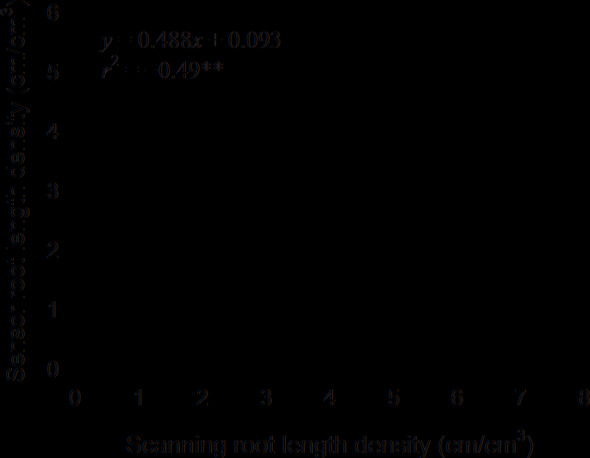
Linear regression of scanning method vs Rootsnap sensor method.

One of the advantages of the Rootsnap sensor is that it can be used to determine changes in root length density dynamically over time. The root length density over time in the subsoil tended to follow the S-shape of a typical growth curve increasing with time (**Figure 7**). This was due to spurts of lateral roots observed in the sideways facing Rootsnap sensor in **Figure 5**. Compared to the scanning method, root length density obtained from the Rootsnap sensor method by using Eq. (3) was significantly correlated with the scanning method (*y* = 0.4663*x*+0.0932, *r* ^2^ = 0.50, p = 0.01) as shown in **Figure 8**.

### Effect of Biochar on Root Traits

The root length density at the 20–50 cm depth at harvest time of maize in 2015, i.e. at maturity, estimated by the Newman counting method is presented in **Table 2**. Only B0 and B3 were analysed at this depth due to the presence of biochar complicating the analysis of roots. Our results indicate that B3 tended to decrease L_V_ in both irrigation schemes although not significantly. Also, no interaction among irrigation level and biochar levels was detected. At the 50–70 cm depth (**Table 3**), biochar treatments, with the exception of B2, tended to decrease L_V_ compared with the control. On the contrary, there was a slight increment with B2. Again, these differences were not significant. The results of 2015 at 50–70 cm depth were compared only between biochar levels due to inadequate number of replicates in the B2 treatment under full irrigation.

**Table 2:** Root length density for B0 and B3 in the 20-50 cm layer in 2015 determined by the Newman method.

Treatment	B0D	B3D	B0FI	B3FI
Root length density (cm/cm^3^) at 20-50 cm	4.38 ± 0.70	3.4 ± 0.44	4.02 ± 0.40	3.66 ± 0.90

Mean values ± standard error (n = 4). Letters not shown when there is no significant difference.

**Table 3:** Root length density and average diameter at 50–70 cm in 2015.

Treatment	B0	B1	B2	B3
Root length density (cm/cm^3^)	2.52 ± 0.20	1.73 ± 0.20	2.75 ± 0.64	2.09 ± 0.29
Average diameter (mm)	0.19 c	0.20 ± bc	0.21 ab	0.22a

Mean values ± standard error (n = 7(B0), 7(B1), 3(B2), 6(B3)).Mean values with no letters in common are significantly different (P < 0.05). Letters not shown when there is no significant difference.

In 2016, biochar application tended to decrease L_V_ at 50–70 cm depth at maturity under both irrigation schemes (**Table 4**). With the exception of B3, all treatments tended to have higher L_V_ under drought than fully irrigated conditions. Under drought, the trend was that of lower L_V_ with increasing biochar level though there was no significant difference between B0D, B1D and B2D. In contrast B3D was significantly lower (P=0.002) than B0D. Under FI, the direction was less clear with B0FI and B3FI being significantly (P ≤ 0.042) higher than B1FI and B2FI. A comparison between the 2 years showed the same trend of lower L_V_ with biochar application when comparing B3 with B0 (compare **Table 3** with **Table 4**) and as well in general appreciably lower L_V_ in 2016 at maturity. The average root diameter in 2015 in the 50–70 cm soil depth (**Table 3**) showed an increase with increasing biochar concentration. The root diameter in B3 (0.22 mm) and B2 (0.21 mm) were significantly greater (P ≤ 0.027) than B0 (0.19 mm). In 2016 the picture was, however, opposite with all biochar treatments having significantly (P=0.003) lower root diameter than the B0 (**Table 4**) under drought, whereas there was no significant effect of biochar level under full irrigation. In B0, full irrigation resulted in significantly lower root diameter than under drought while the root diameter in the biochar treatments did not differ between full irrigation and drought.

**Table 4:** Root length density and average diameter at 50–70 cm for different biochar treatments in 2016.

Treatment	B0D	B1D	B2D	B3D	B0FI	B1FI	B2FI	B3FI	
Root length density (cm/cm^3^)	0.96 ± 0.30a	0.58 ± 0.09ab	0.58 ± 0.10ab	0.36 ± 0.02b	0.82 ± 0.02a	0.53 ± 0.02b	0.52 ± 0.03b	0.71 ± 0.05a	
Average diameter (mm)	0.22 a	0.21 b	0.20 b	0.21 b	0.20	0.20	0.20	0.21	

Mean values ± standard error (n = 4). SE not shown whenless than 0.01.Mean values with no letters in common are significantly different (P < 0.05). Letters not shown when there is no significant difference.

## Discussion

### Images and Video From the Rootsnap Sensor

The root sensor is an easily assembled tool that was used to take maize and grass root images *in situ*. The streamer software on Linux operating system allows automatically capturing and storing of images, which can be accessed remotely. The sensor system seems to have broad applicability also for installation in natural soil, where it might be installed into the side of an excavated wall profile.

### Effect of Biochar on Root Penetration to Subsoil Layer

The average penetration rate of maize roots has been reported to be 2.4–2.5 cm/day (Hsiao et al., 2009). In sandy loam soils, Bengough & Mullins (1988) cited in Bengough and Mullins (1990) found that maize root elongation was reduced by compaction and mechanical impedance to between 50% and 90% of that of control plants grown in loose sieved soils. This shows that maize root penetration is very sensitive to soil physical conditions. In this study, the first root of the control plant at 50 cm depth arrived at day 56 after planting, giving a root penetration rate of approximately 0.8 cm/day, and thus indicating the large extent of mechanical resistance to root penetration plants experienced in these coarse sandy soils. In general, high soil bulk density and water stress increases penetration resistance and slow root elongation (Lampurlanés and Cantero-Martinez, 2003; Bengough et al., 2011). Studies (Abel et al., 2013; Basso et al., 2013) have shown that biochar amendment to sandy soils decreases bulk density and increase water holding capacity. In Danish coarse sand, Bruun et al. (2014) assumed that the addition of biochar might reduce mechanical resistance due to the reduction in subsoil bulk density. A finding that is consistent with our measurement of bulk density, which indicate significant decrease of bulk density at the 20–50 cm layer with increasing biochar application rate (**Table 5**). Further to this, using observations from the Rootsnap sensor, we were able to demonstrate that biochar decreased penetration resistance as evidenced by the shorter time (**Figure 5**) spend by roots to arrive at the 50 cm depth in the B3 treatment compared to the control. The treatment with the highest biochar concentration, B3, showed the lowest bulk density and had the fastest emergence time, suggesting that biochar reduced mechanical resistance in coarse sandy soil. Other factors may however have contributed to this finding. The biochar used contained 2.6% K and thus supplied a large amount of this macronutrient to the plant. Andersen et al. (1992) found that increasing supply of K to barley under field conditions significantly increased the root length density in the subsoil of the same soil type. A meta-analysis by Xiang et al. (2017) found that biochar addition in general increases root biomass, length and surface area in annual crops, consistent with the findings of Abiven et al. (2015) in maize grown in the field. In chickpea Egamberdieva et al. (2019) likewise found increased root mass, but only for hydrothermal biochar amendment and not for a conventionally pyrolysed biochar. As soil dries at the surface, water may be available deeper in the profile (Comas et al., 2013) hence the arrival in the deeper layer may result in better adaptation of biochar plants under drought conditions. Ahmed et al. (2018) found indications of this, as sap flow measurements showed transpiration to be higher in biochar amended plants than in unamended, although soil water content was similar during the second drying cycle of 2015.

**Table 5:** Bulk density measured in 2015.

Bulk density (g/cm^3^)	B0	B1	B2	B3
20–50 cm	1.38 ± 0.01a	1.27 ± 0.01b	1.21 ± 0.01c	1.16 ± 0.01d
50–70 cm	1.36 ± 0.02a	1.29 ± 0.02b	1.23 ± 0.02b	1.17± 0.03c

Mean values ± standard error (n = 4). Mean values with no letters in common are significantly different (P < 0.05). Letters not shown when there is no significant difference.

### Rootsnap Root Length Density Estimates

Overall, L_V_ was under-estimated by the Rootsnap sensor method although it yielded a positive, significant and linear correlation with the scanning method. It should thus be possible to calibrate the method to yield unbiased estimates. Root counting with the Rootsnap sensor resembles the core break method, where a usually vertical soil core is broken to expose a horizontal cross section and roots are counted on both surfaces. This is based on the principle that the same root cannot be exposed on both sides after breakage (Smit et al., 2000). If roots are assumed to grow isotropically (i.e. equally in all directions), anisotropy (A) in Eq. (2) is equal to zero and Eq. (3) simplifies to L_V_ = 2N_A_. However, it has often been found that the proportionality factor is higher than 2 (e.g. Andersen et al., 2013), which thus may be taken as an indication of anisotropic growth. Although our method was designed to take such phenomena into account, the horizontally facing Rootsnap sensor (**Figure 4**) may itself have obstructed roots from reaching it. This seems likely as the backside of the sensor cannot be penetrated by roots and thus will induce a “shadowing” effect, which likely decreased the number of root tips reaching the mesh on the horizontally facing sensor. Due to gravitopism it seems more likely that the correct number of root tips will be counted by the vertically facing sensor.

Different techniques of root length density estimation produce highly variable results and more often than not, are difficult to compare (Pierret et al., 2005). Disparities in results obtained from comparing different root methods have been reported by some studies (Majdi et al., 1992; Wahlström et al., 2015). In their experiment with maize roots Majdi et al. (1992) showed a significant correlation (r = 0.78) between the minirhizotron and the line intercept method for estimating root length density. In contrast, they found no correlation between L_V_ obtained from minirhizotron and washed root analysed using computer image processing. They attributed the higher estimates in L_V_ by the line intercept method to probable overestimation of actual number of intercepts and lower estimates from the computer processing to discarding of smaller root fragments. Wahlström et al. (2015) demonstrated that L_V_ estimates of three methods were in general only weekly correlated except for root washing and scanning versus core break for fodder radish (*r* ^2^ = 0.77) and core break versus minirhizotron for winter wheat (*r* ^2^ = 0.26). The higher estimate by minirhizotron method was attributed to preferential growth along the access tubes.

The overestimation by line intercept and minirhizotron methods presented in the previous paragraph highlights the fact that each method has its own shortcomings. It is important to be aware of the shortcomings of each method and select them based on the root parameters of interest. Our new method yields a significant positive correlation with the scanning method, which shows a potential for determining L_V_ *in situ*. In addition, it presents the opportunity to study natural positions and arrangement of roots i.e. clumping phenomena, which are lost in the root washing method (Judd et al., 2015). Using Eq. (3) in the isotropic case, it is apparent that the sensor area of 12 cm^2^ means that 6 root detections will give an L_V_ equal to 1 cm cm^-3^, which constitute an already high root density for subsoils. Accordingly, sensor estimates of low L_V_ values inherently have a high coefficient of variation. This variation could be reduced by increasing the Rootsnap sensor area, depending on the feasibility of installing more voluminous sensors in a particular application. Nevertheless, the Rootsnap sensor seems unsurpassed for detection of first arrivals of roots to a certain depth avoiding the problems that seems inherent to e.g. the minirhizotron method.

### Effect of Biochar on Subsoil Root Length Density and Diameter

A comparison of L_V_ between the 2 years for all treatments showed lower values in 2016 compared to 2015. This could be due to weather conditions which also resulted in lower aboveground biomass reported by Ahmed et al. (2018). There was no significant difference in L_V_ at 20–50 cm for B0 and B3 under both irrigation schemes in 2015. In addition, there was no significant effect of biochar at the 50–70 cm depth in 2015. The trend in 2016 at the 50–70 cm layer under drought was that of a decrease in L_V_ with increasing biochar level. This could be due to maize root adaptation to drought, which results in the maintenance of root elongation and inhibition of shoot elongation (Saab et al., 1990) in order to maintain an adequate water supply. Thus, the significantly lower RLD in B3 probably indicates that it was the least water stressed. The lack of a clear-cut trend under full irrigation is similar to that observed by Bruun et al. (2014), who reported increases in root density at 40–80 cm depth with 1% and 2% straw biochar. In contrast, application at 4% resulted in decreased density. As soil dries, matric potential becomes more negative and soil strength increases (Bengough et al., 2011). With the tendency of biochar to decrease soil mechanical resistance and increase soil water content, roots under FI may not require adaptation mechanisms such as larger diameter to penetrate the soil, nor more roots to exploit available water. This may explain the lack of significant difference in root diameter under FI and the lower root length densities in the biochar treatments compared with the control.

In drought stressed maize grown in vermiculite, roots showed an increase in length but decrease in diameter (Sharp et al., 1988). In the first year, B2 and B3 significantly increased root diameter compared to B0. The significantly lower diameter in B0 suggests that they experienced the most drought stress. The diameter of roots has also been shown to increase with mechanical resistance (Bengough and Mullins, 1990). Although the soil used here is coarse sand, we do not expect any differences in the mechanical resistance between the control and the biochar treatments at the 50–70 cm depth, as there was no biochar application in this layer. Therefore, we suspect that the decrease in diameter especially for B0 in year 1 was a response to drought. In year 2, however, the soil had been in the pots for a year and frequent irrigation may have caused biochar particles from the 20–50 cm layer to leach into the 50–70 cm layer. As a result, mechanical resistance may have been lesser in the biochar treatments compared with control. Studies on root elongation of cotton as a function of soil strength and soil water content showed root elongation was more sensitive to soil strength than water content (Taylor and Ratliff, 1969 cited in Ball et al., 1994). We therefore speculate that significantly higher diameter in B0D compared to the biochar treatment may be due of higher mechanical resistance.

There is very limited studies on the effect of biochar ageing on root development. The closest comparable study is by Prendergast-Miller et al. (2014), who experimented with fresh and artificially weathered miscanthus and willow biochar. Their results showed a tendency for spring barley roots in weathered biochar-amended soils to have less branched roots than the control and fresh biochar treatments, although this was not significant. Our determination of L_V_ did not show any change in trends with biochar ageing as B0 tended to have higher L_V_ than biochar treatment in both years. However the negative root geotropism in B3 reported by Ahmed et al. (2018) in year 1 was not found in year 2.

## Conclusions

The newly developed Rootsnap sensor presents an easily assembled, cost effective means of monitoring roots *in situ*. The new method was shown to provide estimates of root length density that had a significant positive correlation to the conventional root wash and scanning method. Moreover, temporal changes could be followed and thus e.g. provide important input to dynamic simulation models in which root length density is often a key variable and difficult to obtain. Images obtained from the new method demonstrated that biochar did reduce mechanical resistance in coarse sandy subsoils. The early arrival of roots to the 50 cm depth of up to 9 days before the control, indicate that they may gain access to water available in deeper soil layers that may prevent or delay onset of drought stress. Indeed this phenomena was indicated by sap flow measurements on the maize stems (Ahmed et al., 2018). Maize root respond to drought stress with increased root length density and thinner diameters. This is evidenced by increased root diameter for biochar treatments in the first year and decreased root length density in the second year. There is, however, the need for field studies under both full irrigated and drought conditions. From a methodological perspective, more experiments with the Rootsnap sensor with different crops at different depths and under field conditions are needed to further improve the technique.

## Data Availability Statement

The datasets generated for this study are available on request to the corresponding author.

## Author Contributions

FA: The lead in the planning, experimental set up and writing of the manuscript. EA: Involved in the planning, experimental set up and writing of the manuscript. HL: Involved in the analysis of root samples and reviewing of the manuscript. MA: Involved in the planning, experimental set up and writing of the manuscript.

## Conflict of Interest

The authors declare that the research was conducted in the absence of any commercial or financial relationships that could be construed as a potential conflict of interest.
